# Noninvasive in vivo imaging of macrophages: understanding tumor microenvironments and delivery of therapeutics

**DOI:** 10.1186/s40364-025-00735-9

**Published:** 2025-01-26

**Authors:** Prakash Gangadaran, Akanksha Onkar, Ramya Lakshmi Rajendran, Anshika Goenka, Ji Min Oh, Fatima Khan, ArulJothi Kandasamy Nagarajan, Sathish Muthu, Anand Krishnan, Chae Moon Hong, Byeong-Cheol Ahn

**Affiliations:** 1 BK21 FOUR KNU Convergence Educational Program of Biomedical Sciences for Creative Future Talents, Department of Biomedical Sciences, School of Medicine, Kyungpook National University, Daegu, 41944 Korea; 2 Department of Nuclear Medicine, School of Medicine, Kyungpook National University, Daegu, 41944 Korea; 3 Cardiovascular Research Institute, Kyungpook National University, Daegu, 41944 Republic of Korea; 4 Department of Laboratory Medicine, University of California San Francisco, San Francisco, CA 94143 USA; 5 Department of Hematology and Medical Oncology, Winship Cancer Institute, Emory University, Atlanta, GA 30322 USA; 6 Department of Cancer Biology, Lerner Research Institute, Cleveland Clinic, Cleveland, OH 44195 USA; 7 Department of Genetic Engineering, SRM Institute of Science and Technology, Kattankulathur, Chengalpattu, 603203 Tamilnadu India; 8 Department of Orthopaedics, Government Medical College, Tamil Nadu 639004 Karur, India; 9 Department of Biotechnology, Faculty of Engineering, Karpagam Academy of Higher Education, Tamil Nadu 641021 Coimbatore, India; 10 Precision Medicine and Integrated Nano-Diagnostics (P-MIND) Research Group, Office of the Dean, Faculty of Health Sciences, University of the Free State, Bloemfontein, 9300 South Africa; 11 Department of Nuclear Medicine, School of Medicine, Kyungpook National University, Kyungpook National University Hospital, Daegu, 41944 Korea

**Keywords:** In vivo imaging, Macrophage, Tumors, Optical imaging, MRI, PET, SPECT, Drug resistance

## Abstract

Macrophages are pivotal in the body’s defense and response to inflammation. They are present in significant numbers and are widely implicated in various diseases, including cancer. While molecular and histological techniques have advanced our understanding of macrophage biology, their precise function within the cancerous microenvironments remains underexplored. Enhancing our knowledge of macrophages and the dynamics of their extracellular vesicles (EVs) in cancer development can potentially improve therapeutic management. Notably, macrophages have also been harnessed to deliver drugs. Noninvasive in vivo molecular imaging of macrophages is crucial for investigating intricate cellular processes, comprehending the underlying mechanisms of diseases, tracking cells and EVs’ migration, and devising macrophage-dependent drug-delivery systems in living organisms. Thus, in vivo imaging of macrophages has become an indispensable tool in biomedical research. The integration of multimodal imaging approaches and the continued development of novel contrast agents hold promise for overcoming current limitations and expanding the applications of macrophage imaging. This study comprehensively reviews several methods for labeling macrophages and various imaging modalities, assessing the merits and drawbacks of each approach. The review concludes by offering insights into the applicability of molecular imaging techniques for real time monitoring of macrophages in preclinical and clinical scenarios.

## Introduction

The activity and profile of immune cell infiltrates in tumors significantly influence cancer outcomes. Noninvasive molecular imaging has proven beneficial for a comprehensive understanding of tumor-immune cell interactions. Molecular Imaging utilizes techniques to visualize the cells, either through modification of the cell (i.e. transfection of genetic reporters) or loaded with imaging agents before being injected into subjects. These immune cells then navigate the bloodstream and target specific sites in the tumor microenvironment (TME). Additionally, they can act as therapeutic vehicles due to their intrinsic surface properties, which facilitate chemical conjugation and targeted delivery of therapeutic agents [1–3]. Presently, antitumor immunity in cancer patients is assessed using tissue biopsies and blood biomarkers. However, these invasive methods lack spatial information and do not provide a comprehensive view of the TME, particularly regarding the tumor’s potential heterogeneity. Thus, as an alternative strategy, noninvasive methods for imaging immunoregulatory cells, such as macrophages and cytotoxic immune cells, are being promoted and have shown promise in preclinical and clinical settings [1, 4].

Molecular imaging involves noninvasive monitoring and recording of the biological processes at the cellular and molecular levels in intact living cells [5]. It has been widely used in both animal models and clinical settings. Noninvasive molecular imaging methods include fluorescence, bioluminescence, magnetic resonance, and nuclear imaging. Additionally, when coupled with comprehensive vascular assessment, multimodal imaging provides more accurate information on tumor biology, enhancing the clinical value of molecular imaging [6]. Intriguingly, molecular imaging of the migration and infiltration of tumor-infiltrating immune cells, including cytotoxic T cells, natural killer (NK) cells, and macrophages, has shown success preclinically and clinically, demonstrating great potential in the field [1, 7, 8]. Activated T cells are major contributors to antitumor immunity, making their tracking of particular interest. For example, upregulated surface protein markers (e.g., OX40, ICOS, and CD25) or secreted markers (e.g., IFN-γ and Granzyme B) are attractive targets for the noninvasive imaging of activated T cells [1]. Additionally, optical imaging of NK cells, either transfected with fluorescence or luminescence genes or labeled with dyes, has facilitated tracking their migration and infiltration in tumors [5, 7].

Macrophages, the predominant myeloid population of tumor-infiltrating innate immune cells, are categorized into tumor-promoting and tumor-inhibiting macrophages. Both categories have been imaged by targeting the CD11b^+^ myeloid cells in the TME [9]. The tumor-promoting population, known as tumor-associated macrophages (TAMs), is the most abundant myeloid population infiltrating the TME, and their abundance correlates with poor patient survival in most cancers [9–11]. TAMs have been implicated in developing resistance to therapies, including resistance to immune checkpoint inhibitors in several cancer types [12, 13]. Hence, tracking macrophages and their function within tumors is crucial for comprehending the TME, elucidating underlying disease mechanisms, and designing potential macrophage-based therapies and drug-delivery systems.

Extracellular vesicles (EVs) are nano-sized membranous vesicles released by almost all cells into extracellular space and invitro into culture media. EVs are generally classified into exosome (small EVs), microvesicles and apoptotic bodies based their biogenesis. Exosomes are released into extracellular space upon fusion of multivesicular bodies and the plasma membrane are released from the surface of cells. Whereas apoptotic bodies released during the cells undergoing apoptosis. EVs plays a vital role in cell-to-cell communication in adjacent and distance cells as they carry various biologically active materials such as lipids, proteins, and nucleic acids [14, 15]. Recent evidence indicates that EVs play a crucial role in key physiological and pathological processes, including cellular homeostasis, infection, and cancer. The growing recognition of EVs as potential biomarkers and therapeutic tools has sparked increased interest in their study [14, 16, 17].

We aim to delve into recent findings regarding the association of TAMs with cancer pathology and progression and their utilization in drug-delivery strategies. Barone et al. [18] focused mostly on the EV categories that are utilized for cancer research and TAM-EV based nanomedicines for cancer treatment. In current review, we emphasize the various noninvasive methods for imaging macrophages and their EVs in cancer. We also portray how these methods hold tremendous potential for broad clinical applications and provide valuable insights into characterizing biomarkers and therapeutic targets. In summary, this review offers a comprehensive analysis of preclinical theranostic approaches for TAMs, highlighting diverse imaging strategies and their clinical relevance in ongoing human trials. The knowledge gained would help understand the immunosuppressive effects exerted by TAMs on cytotoxic immune cells, thereby contributing to the battle against tumor progression.

## Macrophages

Macrophages are multifunctional immune cells found in mammalian tissues. Initially described by Elie Metchnikoff as phagocytic cells [19], early research focused on their roles in host defense against infections, regulating housekeeping genes to remove apoptotic cells and extracellular matrix (ECM) remodeling [20]. However, recent studies have expanded our understanding of macrophages, highlighting their generic roles in metabolism, tissue homeostasis, and development [21, 22]. Tissue-resident macrophages are known to be involved in the development of organs such as the brain, bone, and ovaries, as well as in tissue repair by sensing damage signals [20, 23]. They release growth factors that aid in functions like neuronal patterning, branching morphogenesis, bone morphogenesis, angiogenesis, and adipose tissue formation [23]. In addition to their physiological roles, macrophages contribute to the pathology of various diseases, including osteoporosis, atherosclerosis, fibrosis, and cancer [20]. While macrophage activation through cytokines and bacterial products was initially found to kill cancer cells, later studies revealed that macrophages are often present in leukocyte infiltrates within tumor tissues. Upon interaction with the TME, these infiltrates are driven toward an immunosuppressive TAM phenotype, facilitating cancer progression and therapy resistance [8, 24, 25].

### Classification of macrophages

**Fig. 1:**
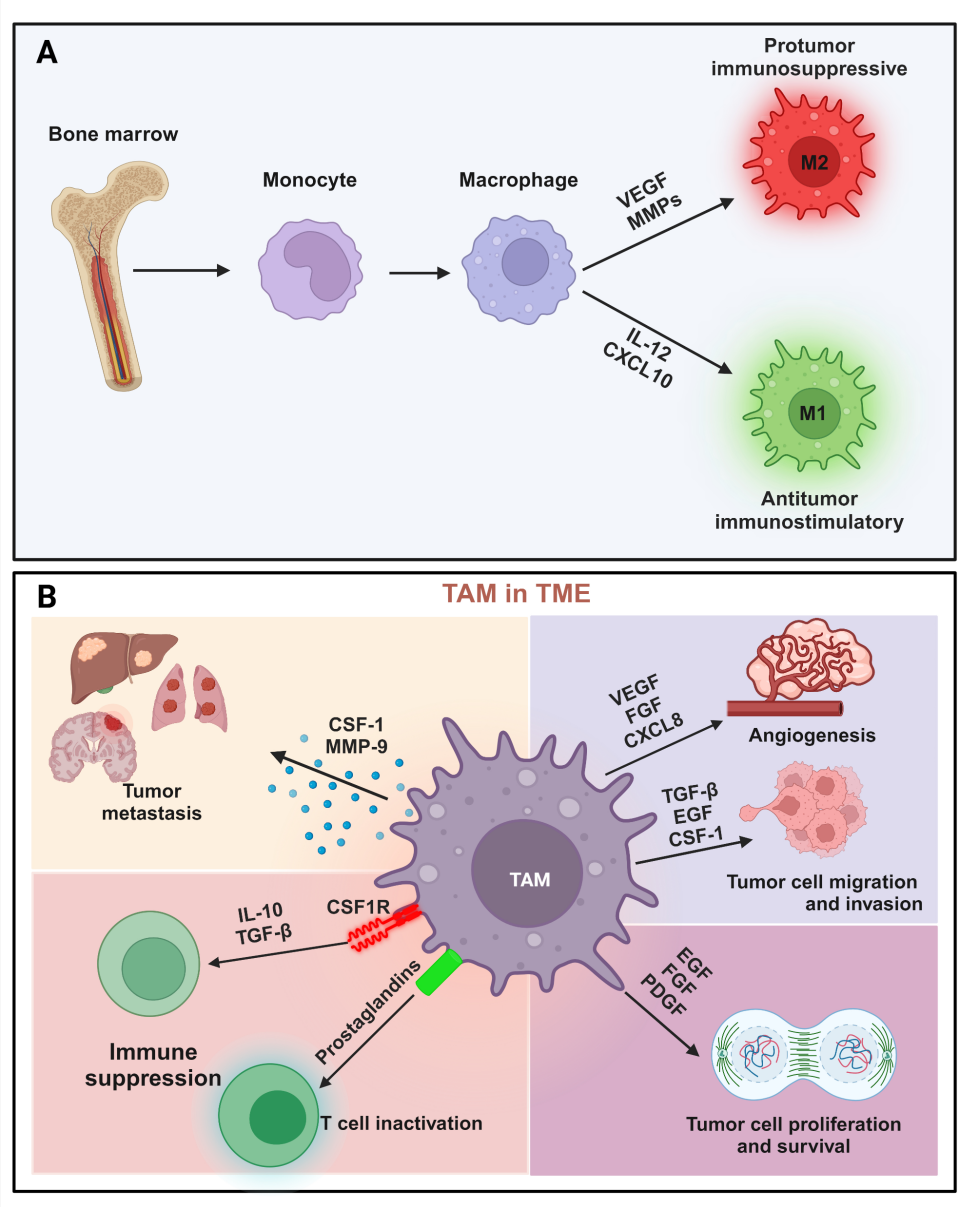
Types of macrophages and role of TAMs in the TME. (**A**) Macrophages polarize into M1 and M2 subtypes, which arise from the bone marrow. Tumor-associated macrophages (TAMs) participate in cancer progression. (**B**) Schematic represents the impact of TAMs in promoting tumor cell metastasis, angiogenesis, T-cell inactivation, epithelial-mesenchymal transition, invasion, and migration. TAMs promote tumorigenesis by secreting some factors and expressing some proteins. CSF-1: colony-stimulating factor-1; MMPs: matrix metalloproteinases; EGF: epidermal growth factor; FGF: fibroblast growth factor; VEGF: vascular endothelial growth factor; PDGF: platelet-derived growth factor. Created with BioRender.com

Macrophages are classified into two main categories: macrophage 1 (M1) and macrophage 2 (M2). These classifications reflect the diversity of macrophages representing the extreme states of in vitro polarization [26]. This categorization has also been adopted to classify macrophages in vivo (Fig. 1A). Treatment with proinflammatory cytokines (e.g., TNF and interferons) or bacterial products (e.g., lipopolysaccharide) induces the polarization of macrophages to an M1 phenotype. Meanwhile, immunoregulatory cytokines such as TGFβ, IL-4, and IL-10 induces an M2 phenotype in the macrophages. This M2 polarization promotes the secretion of proangiogenic factors (e.g., VEGF) and tissue-remodeling enzymes (e.g., matrix metalloproteinases), which facilitate tumor progression [27–29]. In contrast, M1 macrophages release anti-angiogenic factors, such as IL-12 and CXCL10, and are associated with antitumor immunity [30].

Interestingly, TAMs do not strictly conform to the M1 and M2 phenotypes. Single-cell sequencing has been employed to address this limitation, allowing for a more detailed classification of macrophages into additional subtypes: M2a, M2b, M2c, and M2d [31–33]. Despite the different subtypes’ characterization, understanding each subtype’s precise function in tumor survival and progression remains challenging [34]. Moreover, TAM subpopulations often coexpress both M1 and M2 gene signatures, underscoring the heterogeneity of TAMs [35], thereby suggesting new directions for targeted therapy.

### TAMs

As mentioned above, TAMs are significant components of the TME and are associated with poor prognosis and drug resistance in tumors [36]. In patients with classic Hodgkin’s lymphoma, an increase in TAMs is strongly associated with shortened survival, highlighting TAMs as biomarkers for risk stratification [37]. Tumor cells secrete growth factors and cytokines that attract macrophages and induce them into a protumorigenic profile, contributing to poor clinical outcomes in various cancers [38, 39].

TAMs are derived from two major macrophage populations. One population consists of macrophages that differentiate from yolk sac–derived precursors, which can self-renew during a steady state and an infection. The other population originates from bone marrow-derived monocytes (BMDMs), which give rise to macrophages in the intestine and dermis [40]. For example, in glioblastoma, a grade IV lethal form of brain tumor, TAMs are primarily derived from monocytes that extravasate from the blood circulation and infiltrate the inflamed brain tissue, where they were differentiated from bone marrow-derived macrophages [13, 41]. However, understanding the specific origin of macrophages for different cancer subtypes remains limited [40].

Activated TAMs can produce various factors that generate the hallmarks of cancer, including inducing angiogenesis, sustaining proliferative signaling, disrupting the immune system, evading growth suppression, enhancing metastasis and invasion, and suppressing cell death pathways [42]. For instance, cancer cells secrete succinate to polarize macrophages into TAMs, promoting cancer cell migration, invasion, and metastasis through the PI3K-HIFα axis [43]. Similarly, the secretion of the allergic mediator, histamine, by cancer cells and the neurotransmitter GABA by B cells contributes to the generation of tumor-promoting TAMs [44, 45]. Additionally, beyond polarization, cancer cells stimulate TAM amplification by secreting colony-stimulating factor 1 (CSF1, a key regulator that sustains the protumorigenic functions of TAMs), the E3 ligase Cop1, and the metabolite β-glucosylceramide, all of which enhance TAM activity [46–48]. Zhu et al.. recently reviewed the pivotal role of macrophages in the innate immune system and the TME, particularly in thyroid cancer (TC). Their review highlights the dual nature of TAMs in TC progression, examining their polarization, gene mutations, and M2-like TAM-centered therapeutic strategies [49]. Finally, intravital imaging studies have demonstrated long-term physical interactions exist between TAMs and CD8^+^ effector T, leading to T-cell exhaustion [50] (Fig. 1B).

## Macrophages and drug delivery

Due to the phagocytic activity of macrophages, their tumor homing capability, and their efficacy in killing cancer cells, macrophages especially the M1 subtype, have been widely exploited as carriers or vehicles for drug delivery [51, 52]. These drug-delivery systems utilize the surface protein markers of macrophages to target cancers. Apart from the macrophages themselves, macrophage-derived EVs, the secretory vesicles released from macrophages, play an important role in intercellular communication and are broadly used for drug-delivery vehicles in cancerous diseases. EVs in general are classified into the following categories based on their size: (i) exosomes (30–150 nm), (ii) microvesicles or ectosomes (50 nm–1 μm), and (iii) apoptotic bodies (50 nm–5 μm) [53, 54]. Among these, exosomes originate from late endosomes and are secreted by the fusion of late endosomes with the plasma membrane [55]. Therefore, macrophage derived EVs, especially the exosomes, inherit the phenotypic and functional properties of their parent macrophage subtype, and could be used in studying TAM function in the TME and in delivering therapeutics. Additionally, macrophage membranes have been used to coat nanoparticles directly, which serve as anticancer drug-delivery vehicles [53].

### Macrophage-mediated drug delivery

As previously stated, macrophages are efficient and versatile carriers for anticancer drugs. They can circulate in the bloodstream alongside red blood cells and neutrophils and target cancer cells by binding their α4β1 integrins to vascular cell adhesion molecule-1 on cancer cells. M1-like macrophages derived from BMDMs and the macrophage cell line RAW264.7 are widely used for tumor-targeting applications [53, 56]. These direct carriers of anticancer drugs are generated by incubating the macrophages with the drugs. In one study, doxorubicin (DOX)-loaded RAW 264.7 macrophages prolonged the survival of 4T1 tumor-bearing mice, *with DOX-loading* not significantly affecting macrophage viability and function [57]. Additionally, DOX-loaded M1 macrophages transferred DOX to ovarian cancer cells through a tunneling nanotube pathway, similar to virus transfer, which inhibited tumor invasion more efficiently than liposome-DOX [58]. Despite these benefits, the use of RAW 264.7 cells remains controversial due to their transformed nature, which means they lack some of the phenotypical and functional characteristics of primary macrophages. Moreover, using primary macrophages presents its challenges, including the potential for inflammation, development of a pro-tumorigenic phenotype leading to immune tolerance, and possible off-target effects [59–61].

**Fig. 2:**
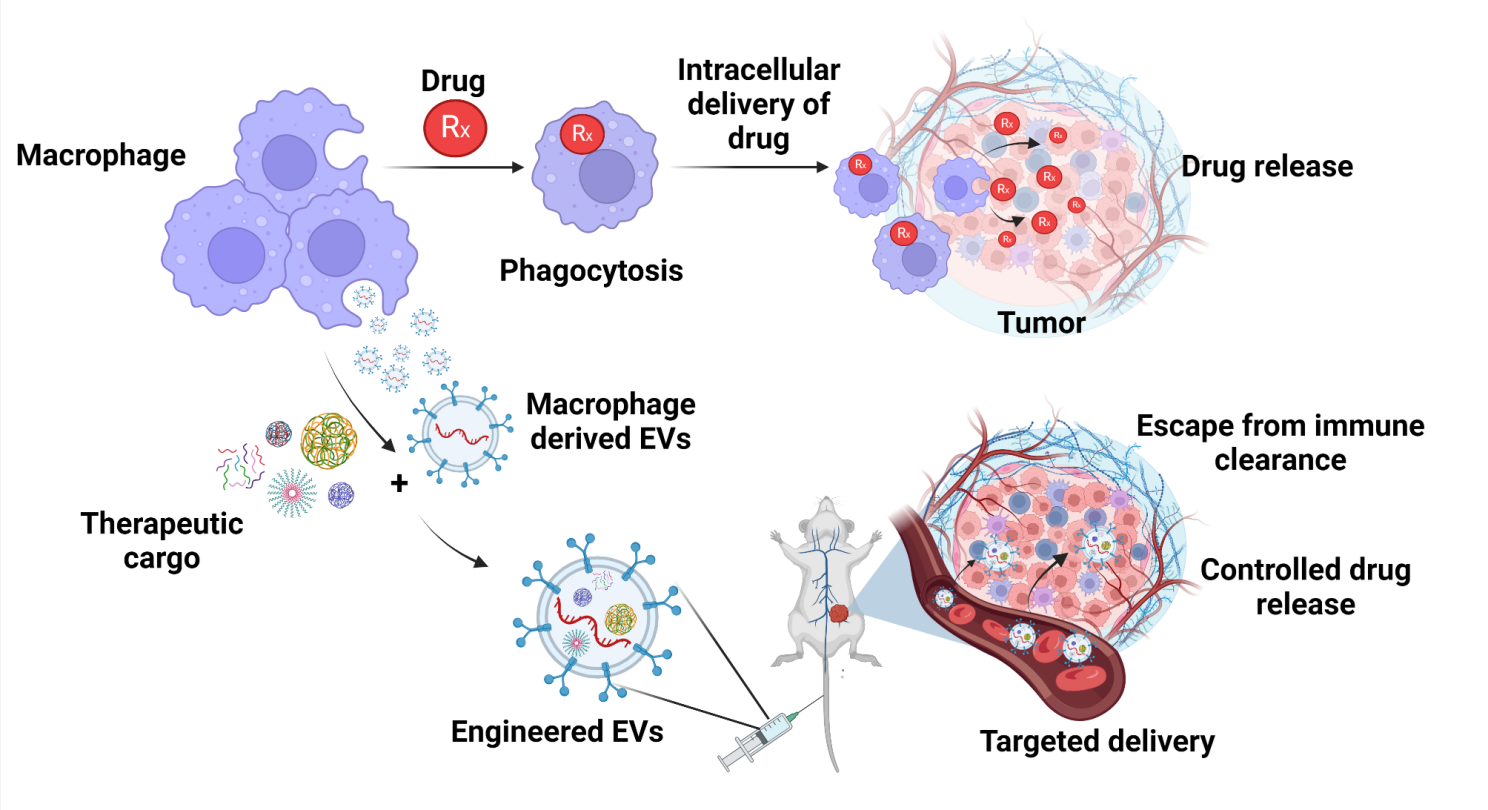
Drug delivery using macrophages or their EVs. Recent understanding of macrophages seems diverse in terms of functions in many diseases. Macrophages have gained increasing interest as critical therapeutic targets. When developing drug carriers, understanding their biological roles in biodistribution, cellular uptake, intracellular trafficking, and drug release is essential for efficient targeting. Macrophage-derived EVs also play a vital role in tumor progression. Their use as natural nanovesicles for therapeutic drug loading has many benefits in blood-brain barrier penetration, whereas other synthetic nanoparticles showed failed blood-brain barrier penetration. Created with BioRender.com

Besides the mentioned drawbacks, when macrophages are used as direct carriers, the cytotoxic effects of drugs on macrophages remain a bottleneck and require monitoring [53]. As a result, most applications use macrophages as indirect carriers wherein nanoparticles (NPs) containing drugs are loaded onto macrophages to help reduce drug toxicity and increase the drug dose. For example, macrophages loaded with N-succinyl-N′-octyl chitosan (SOC)-paclitaxel (PTX) demonstrated higher therapeutic efficiency than PTX-loaded macrophages due to their higher drug-loading efficiency [62]. Moreover, NP-loaded M1-like macrophages could cross the blood-brain barrier, showing favorable brain distribution and enhanced survival in glioma-bearing mice treated with DOX-loaded M1 macrophages [63]. The macrophages endocytosed and released the loaded drugs; however, most drugs were degraded during this process, which remained the study’s limitation. This issue was potentially overcome by Doshi et al., who developed phagocytosis-resistant backpacks, wherein loaded microparticles were attached to the macrophages’ surface rather than the drug being engulfed [64] (Fig. 2).

### Macrophage-derived EVs mediated drug delivery

As described earlier, M1-like macrophage-derived exosomes (M1-exos) have surface membrane properties like their parent macrophages and could be used as therapeutic anticancer agents. For instance, a nanoformulation of M1-exos loaded with PTX efficiently treated drug-resistant tumors [65]. However, this study used exosomes from RAW 264.7 cells. It remains to be seen if using macrophage exosomes derived from BMDMs would yield better outcomes. Surface receptors could also be targeted, for example, M2-polarized TAMs express higher levels of the interleukin-4 receptor (IL4R) than M1-polarized macrophages [66] In a study by Gunassekaran et al., engineered M1-derived exosomes transfected with NF-κB p50 siRNA and miR-511–3p, termed Exo(si/mi), were utilized to promote M1 polarization and target IL4R. Whole-body fluorescence imaging revealed that DiD-labeled IL4R-Exo(si/mi) exosomes successfully reprogrammed tumor-associated macrophages (TAMs) into an M1 phenotype, thereby inhibiting tumor growth. IL4R-Exo(si/mi) exosomes were efficiently internalized by M2 macrophages, leading to a decrease in M2 markers, an increase in M1 markers, and more efficient suppression of target genes compared to control exosomes. Furthermore, imaging demonstrated that IL4R-Exo(si/mi) exosomes accumulated in tumors more effectively than untargeted exosomes. Notably, systemic IL4R-Exo(si/mi) administration hindered tumor growth, lowered M2 cytokines and immune-suppressive cell levels, and elevated M1 cytokines and immune-stimulatory cell levels, significantly outperforming control exosomes. Despite not thoroughly addressing the polarization mechanisms and their effects on the TME, the study highlights IL4R-Exo(si/mi) as a promising cancer immunotherapy strategy [67].

Aside from acting as drug carriers, M1-exos release antitumor cytokines, enhancing their cytotoxic effects [68]. M1-exos can also serve as adjuvants for cancer vaccines; however, the mechanistic understanding behind this remains limited [69]. Exosome-mediated therapy in tumors also faces cost-benefit due to the low yield. To address this, exosome-mimetic nanovesicles have been developed to overcome the low yield problem in exosome purification. In a study by Choo et al., such M1-exo-mimetics polarized M2-like macrophages to an M1-like phenotype, thereby enhancing the efficacy of anti-programmed death-ligand 1 (PD-L1) therapies [70]. Taken together, macrophage-derived exosomes target malignant tumors and modulate the immunosuppressive TME [53] (Fig. 2).

## Noninvasive in vivo imaging modalities: monitoring macrophage migration and infiltration into tumors

The utilization of light for visualizing cells and tissues has been a consistently insightful and straightforward method in basic research and medical diagnostic imaging. In vivo monitoring of macrophage migration and infiltration into tumors can be achieved through the various noninvasive molecular imaging methods discussed below. These techniques facilitate visualization and quantification of the behavior and dynamics of TAMs in the TME, providing valuable insights into tumor progression and therapeutic responses.

### Fluorescence imaging

Over the past decade, significant advancements in the engineering and application of fluorescent proteins have expanded their utility in imaging, enabling more precise visualization of biological processes [71, 72]. These proteins have revolutionized the ability to track and study cellular and molecular processes in real-time, offering unprecedented insights into biological systems. In vivo fluorescent imaging with living organisms mirrors the principles of fluorescence microscopy [73], which involves transducing cells of interest with green or red fluorescent proteins or reporter genes for live imaging [74]. Additionally, fluorescent agents such as dyes Cyanine 5, Cyanine 7, and DiR: 1,1’-Dioctadecyl-3,3,3’,3’-tetramethylindotricarbocyanine iodide, with appropriate emission wavelength and photostability, are employed for in vivo cell imaging investigation [75, 76]. Overall, fluorescence imaging offers comparatively lower expenses and high spatial resolution, which is particularly evident when investigating reporter gene systems in small animals like rodents. Furthermore, unlike bioluminescence imaging (discussed below), fluorescence imaging does not require a substrate. However, its sensitivity is compromised by autofluorescence issues [77] (Table 1). Fluorescence imaging techniques are widely used to track TAMs in cancer studies. A study by Sun et al. conducted fluorescent imaging of TAM in breast tumor-bearing mice, using CD206 antibody-coated NIR-emitting fluorescent dye (NIRF) and dye-IgG as controls. Both probes were intravenously injected into the mice. In vivo NIRF imaging revealed that signals were present as early as 2 h post-injection and continued to be detecTables 8 and 24, and 48 h., the time point at which TAMs were detected in the tumors (Table 2). While the control dye-IgG was also detected in the tumors at all the mentioned time points, it was present at significantly lower levels. Ex vivo imaging further confirmed a two-fold greater signal in the tumor following NIRF-CD206 injection than dye-IgG. Additionally, the NIRF-CD206 signal colocalized with F4/80^+^ TAMs, unlike the controls. Therefore, the study showed that CD206-targeted molecular imaging could sensitively detect the dynamic changes in TAMs. A similar approach was utilized in the study by Zhang et al., where an antibody of CD206-NIRF dye was used to visualize the TAM in the mouse breast tumor. The CD206-NIRF dye was intravenously injected into the mice and imaged after 24 h. In vivo fluorescent imaging showed a significant dye accumulation in the tumor region and lymph node (LN) metastasis compared to the free dye. Thus, this study also confirmed that CD206-targeted imaging can sensitively detect TAMs in the tumors and LN metastasis [112] (Table 2). However, given the heterogeneity of TAMs within and across tumors, the technique utilized in the abovementioned studies offers limited potential for clinical translation. Safety of most of fluorescent dyes (except indocyanine green) is not validated for human use. Moreover, shared markers between immune cells make the method susceptible to a lack of sensitivity.

**Table 1:** Different imaging modalities and their use in assessing tumor microenvironment phenotypes

Imaging modality	Acronym	Contrast agent categories	Advantages	Disadvantages	Tumor phenotype evaluation	Clinical application	References
Fluorescence	FLI	Fluorescent dyes and molecules Light sensitive nanoparticles Quantum dots Photoacoustic contrast agents	• High sensitivity (*invitro*) and specificity • Moderate sensitivity (In vivo) • Real-time imaging • Incorporation of multiple colors • Ease of use and accessibility • Easy sample preparation, Cost-effective	• Limited tissue penetration • Low image quality due to scattering and absorption • Low signal to noise ratio due to autofluorescence • Photobleaching and phototoxicity • Limited resolution • Limited clinical use	Macro/microstructure analysis Detection of specific tumor receptors, proteins, or antigens Analysis of TME heterogeneity using specific markers for cell types	Limited	[78–82]
Bioluminescence	BLI	Luciferases and luciferins Luciferase substrate pairs Synthetic bioluminescent probes Calcium and pH sensitive probes	• High sensitivity (*invitro*) • and specificity • Moderate sensitivity (In vivo) • Real-time imaging • Minimal photobleaching and phototoxicity • Multiplexing capability • Cost-effective	• Limited tissue penetration • Limited imaging depth Dependency on reporter gene expression • Requirement of substrate administration • Limited clinical use • Potential antigenicity of the enzymes	Macro/microstructure analysis Detection of specific tumor receptors, proteins, or antigens Analysis of TME heterogeneity using specific markers for cell types	Limited	[81–89]
Magnetic Resonance Imaging	MRI	Gadolinium based contrast agents Iron-oxide nanoparticles Manganese based contrast agents Hyperpolarized agents ¹⁹Flourine based contrast agents	• Excellent tissue penetration • High resolution due to superior soft tissue contrast • Used non-ionizing radiations • Functional imaging for e.g. fMRI • Broad clinical use	• High cost and limited availability • Time consuming • Susceptible to artifacts due to motion sensitivity • Risks in patients with contrast agents • Less effective for calcified tissues like bone • Less sensitivity	Analysis of TME and its cellular density Proliferation analysis Analysis of apoptosis and necrosis Macro/Microstructure analysis	Yes	[81, 82, 90–96]
Magnetic Resonance Spectroscopy	MRS	Paramagnetic contrast agents Oxygen-sensitive agents pH sensitive agents Enzyme-responsive agents	• Non-invasive • In vivo biochemical insights • Early disease detection • Quantitative in nature • No ionizing radiation • Complementary to MRI	• Low sensitivity • Complex synthesis of probes • Short half-life of the probes	Metabolic and microenvironment phenotypes Analysis of tumor heterogeneity Response to therapy	Yes	[97–100]
Magnetic Particle Imaging	MPI	Iron oxide nanoparticles such as magnetite and maghemite	• High-sensitivity, minimal background noise, and superior contrast • Real-time and quantitative imaging • No exposure to ionizing radiations • High scalability • Minimal interference with MRI • No depth limitation	• Limited spatial resolution • Dependance on synthesis and quality of nanoparticle contrast agents • Requirement of specialized equipment’s for imaging • Potential toxicity from non-biocompatible contrast agents • Lack of anatomical imaging • Limited clinical availability	Tumor localization, size, vascularity and perfusion Tumor targeting and analysis of TME in terms of hypoxia, pH or acidity Metastasis detection	Yes	[101–105]
Positron Emission Tomography/ Single Photon Emission Computed Tomography	PET/SPECT	Glucose metabolism tracers Neurotransmitter and neuroreceptor tracers Perfusion and Hypoxia tracers Amino acid, protein, and DNA synthesis tracers Inflammation and infection tracers Heavy metal labeled agents (SPECT)	• High sensitivity • Functional whole-body imaging possible • High spatial resolution • High tissue penetration • Longer half-life of radiotracers	• Ionizing radiation exposure • Limited availability • Complex radiotracer development • Longer imaging times Provides 2D information if not used along MRI	Analyzing metabolic reprogramming Analysis of conditions like hypoxia/oxygenation Analysis of tumor proliferation Detection and analysis of tumor-related proteins and antigens Analysis of angiogenesis	Yes	[81, 82, 106–111]

FLI: Fluorescence Imaging; BLI: Bioluminescence Imaging; MRI: Magnetic Resonance Imaging; MRS: Magnetic Resonance Spectroscopy; MPI: Magnetic Particle Imaging; PET: Positron Emission Tomography; SPECT: Single Photon Emission Computed Tomography; TME: Tumor Microenvironment; fMRI: Functional Magnetic Resonance Imaging; ¹⁹F MRI: Fluorine-19 Magnetic Resonance Imaging; 2D: Two-Dimensional; pH: Power of Hydrogen (acidity/basicity measurement)

**Table 2:** Molecular imaging of in vivo monitoring of macrophage migration and infiltration into tumors

Imaging	Imaging modality	Labeling Agent	Cell type	Subject	Injection route		Duration	Tumor	Ref.
					Agent	Macrophage			
FLI	FLI	CD206-targeting NIRF	TAM	BALB/c mice	IV	-	2, 8, 24, and 48 h	Mouse breast cancer	[171]
	FLI	BMV083-Cy5	TAM	BALB/c mice	IV	-	10 h	Mouse breast cancer	[113]
	FLI	Deoxymannose-Cy7	TAM	BALB/c nude mice	IV	-	2, 4, 6, and 8 h	Human hepatoma cells	[117]
	FLI	Dye-αCD206	TAM	BALB/c mice	IV	-	24 h	Mouse breast cancer	[112]
	FLI	DiR	BMC2	C57BL/6 mice	-	IV	72 h	Mouse melanoma cells	[76]
BLI	BLI	Effluc	RAW 264.7 cells	BALB/c nude mice	-	SC	0 and 7 days	Mouse colon cancer	[128]
	BLI	Effluc	RAW 264.7 cells	BALB/c nude mice	-	IP	1–4 days	Mouse colon cancer	[128]
MRI	MRI	FIONs	Peritoneal macrophage	BALB/c nude mice		IV	24 h	Mouse melanoma tumor, metastatic lymph nodes	[135]
	MRI	PG-Gd-NIR813	TAM	SD nude rat	IV	-	0 and 48 h	Rat glioma	[116]
	MRI	Ferumoxytol	TAM	Human	IV	-	24 h	Human lymphomas or sarcomas	[139]
	MRI	Ferumoxytol, P904 or P1133.	TAM	FVB mice	IV	-	1 and 24 h	Mouse mammary adenocarcinomas	[140]
	MRI	Dextran coated SPIONs	TAM	FVB/N mice	IV	-	24 h	Mouse mammary adenocarcinomas	[141]
	MRI	Ferumoxytol	TAM	C57BL/6 or BALB/c nude	IV	-	24 h	Mouse lung carcinoma	[162]
	MRI	¹⁹F	TAM (metastasis-associated macrophages)	Female BALB/c mice	IV	-	12, 24, 36, 48 and 60 h	Mouse breast cancer	[142]
	MRI and MRS	Nitric oxide-targeting USPIO	TAM	BALB/c	IV	-	1, 6, 12, 24, and 48 h	Mouse breast cancer	[146]
	MRI and MPI	Ferumoxytol and Ferucarbotran	TAM	Female BALB/c mice	IV	Orthotopic injection in mammary fat pad	24 h	Mouse breast cancer	[105]
NI	PET/CT	^124^I-Au@AuCBs	RAW 264.7 cells	BALB/c nude mice	-	IT	1 and 9 days	Mouse colon cancer	[153]
	PET/CT	^89^Zr-PL-HDL & ^89^Zr-AI-HDL	TAM	C57BL/6 mice	IV	-	24 h	Mouse breast cancer	[155]
	PET	Mannose coated ^64^Cu liposomes	TAM	FVB mice	IV	-	6 h	NaCl-induced lung tumor	[160]
	PET/CT	^18^F-FB-antiMMR sdAb	TAM	C57BL/6 mice	IV	-	60 and 180 min	Mouse lung carcinoma	[158]
	SPECT-mCT	^99m^Tc-labeled antiMMR nanobodies	TAM	WT or MMR-KO-mice	IV	-	3 h	Mouse lung carcinoma	[161]
	PET/CT	^64^Cu- and VT680-labeled Macrin	TAM	C57BL/6 mice	IV	-	24 h	Mouse Colon Adenocarcinoma/Mouse lung adenocarcinoma	[159]
	PET/CT	FDG	TAM	Human	IV	-	-	Human non–small cell lung cancer	[162]
	PET	FDG	TAM	C57BL/6 or BALB/c nude	IV	-	24 h	Mouse Lung carcinoma	[162]
	SPECT/CT	^125^I-αCD206	TAM	BALB/c mice	IV	-	24 h	Mouse breast cancer	[112]

FLI - Fluorescence Imaging, CD206 - Cluster of Differentiation 206 (a cell surface receptor), NIRF - Near-Infrared Fluorescence, TAM - Tumor-Associated Macrophages, BALB/c mice - A strain of laboratory mice, IV - Intravenous (administration route), BMV083-Cy5 - A specific compound labeled with Cy5 fluorophore, Deoxymannose-Cy7 - A compound labeled with Cy7 fluorophore, Dye-αCD206 - A dye-labeled antibody targeting CD206, DiR - A lipophilic near-infrared dye, BMC2 - Engineered macrophage expressing luciferase, C57BL/6 mice - Another strain of laboratory mice, BLI - Bioluminescence Imaging, Effluc - Firefly Luciferase gene, RAW 264.7 cells - A mouse macrophage cell line, SC - Subcutaneous (administration route), IP - Intraperitoneal (administration route), MRI - Magnetic Resonance Imaging, FIONs - Functionalized Iron Oxide Nanoparticles, PG, Gd-NIR813 - A specific contrast agent labeled with Gd and NIR813 fluorophore, Ferumoxytol - An iron supplement used as a contrast agent, P904 or P1133 - Specific compounds used in imaging, Dextran coated SPIONs - Dextran-coated Superparamagnetic Iron Oxide Nanoparticles, NI - Nuclear Imaging, PET/CT - Positron Emission Tomography/Computed Tomography, ^124^I-Au@AuCBs - Gold-labeled carbon black nanoparticles labeled with iodine-124, ^89^Zr-PL-HDL & ^89^Zr-AI-HDL - Zirconium-labeled phospholipid and apolipoprotein A-I high-density lipoprotein, ^64^Cu - Copper-64, ^18^F-FB-antiMMR sdAb - Fluorine-18-labeled single-domain antibody against Macrophage Mannose Receptor, SPECT-mCT - Single Photon Emission Computed Tomography/micro Computed Tomography, ^99m^Tc-labeled antiMMR nanobodies - Technetium-99m-labeled nanobodies against Macrophage Mannose Receptor, VT680-labeled Macrin - VT680 fluorophore-labeled Macrophage Receptor with Collagenous Structure, FDG – Fluorodeoxyglucose, ^125^I-αCD206 - Iodine-125-labeled alpha Cluster of Differentiation 206 antibody

Another study by Verdoes et al. employed a nonpeptidic cathepsin S activity-based probe with Cy5 (BMV083-Cy5) in breast orthotopic tumor-bearing mice for TAM identification. The BMV083-Cy5 probe was intravenously injected into the orthotopic tumor-bearing mice, and an in vivo fluorescent signal was detected in the tumor 10 h post-injection (Table 2). Since fluorescence imaging suffers from the limitation of lower sensitivity than bioluminescence (Table 1), the authors labeled the 4T1 tumor cells with luc-GFP to demarcate the tumor boundary using luciferase bioluminescence, while the localization of the BMV083 was determined by Cy5 fluorescence. Interestingly, the BMV083 signal primarily colocalized with the F4/80^+^ macrophages, establishing TAMs as the significant source of cysteine cathepsin activity in the TME [113]. However, the authors identified F4/80^hi^ as the primary BMV083^+^ cells and classified them M2-macrophages. This classification remains a shortcoming given the redundancy of F4/80 expression on other macrophage subtypes, including M1-macrophages [114]. A potential method to overcome such limitation could involve conjugating fluorescent probes to antibodies or ligands specific to macrophage surface markers or TAM-related antigens, allowing for highly specific targeting [112, 115, 116]. Another study by Zambito et al. utilized a similar combination of fluorescence and bioluminescence imaging to track TAMs. The authors used engineered macrophages (BMC2) as sensors for metastatic melanoma, demonstrated through dual-color in vivo imaging. Macrophages expressing the green click green luciferase and labelled with the NIR dye were attracted to melanoma cells expressing near-infrared click beetle luciferase, detectable through real-time imaging up to 72 h after injection (Table 2). The study shows potential in early detection and effective treatment strategy for melanoma, which usually suffers from poor prognosis upon metastasis. Thus, optical imaging can potentially detect noninvasive metastatic melanoma using circulating macrophages [76].

In human hepatoma tumor-bearing mice, the in vivo fluorescent imaging of TAM was conducted by intravenously injecting deoxy mannose (DM), a high-affinity ligand of mannose receptor, labeled with the NIR dye cyanine 7 (Cy7). The authors observed specific signals in the tumor as early as 1 h. post-injection, which gradually stabilized at 2, 4, 6, and 8 h (Table 2), and the fluorescence slightly decreased over time. Ex vivo imaging further confirmed the presence of TAMs in the tumors using DM-Cy7 [117]. Now, mannose receptors may also be present on other immune cells or cell types within the TME [118]. Thus, the specificity of mannose labeling for TAMs must be carefully validated when such methods are translated into clinical studies.

Thus, fluorescence imaging alone, and in combination with bioluminescence, offers a powerful tool for studying TAMs in the TME and in monitoring treatment outcomes. However, the method suffers from limitations of photobleaching, which shortens the duration of in vivo imaging experiments [5, 7, 119, 120]. Additionally, tissues may exhibit autofluorescence, which can interfere with specific signal detection [78, 121, 122] (Table 1).

### Bioluminescence imaging

Bioluminescence imaging operates through light production from enzymatic oxidation reactions involving luciferases such as Firefly luciferase (Fluc), Renilla luciferase (Rluc), Gaussia luciferase (Gluc), and their substrates like D-Luciferin or coelenterazine [123]. These luciferase enzymes can function as molecular reporting devices when introduced into a biological system, typically through transfection with their encoding genes [124, 125]. In contrast to fluorescence imaging, bioluminescence doesn’t rely on an external light source. This, coupled with the absence of endogenous bioluminescence in tissues, allows for greater sensitivities and higher signal-to-background ratios compared to fluorescence techniques [83, 126, 127]. However, as mentioned above, the clinical translatability of bioluminescence may be limited by substrate requirements and the spatial resolution for detailed cellular imaging is compromised compared to fluorescence imaging techniques. Moreover, enzymes such as luciferases and their cofactors, which are commonly used in the bioluminescence imaging modality, are of foreign origin and can trigger an immune response in the host [85–87]. This antigenic stimulation may result in the production of neutralizing antibodies, potentially causing hypersensitivity and inflammation, thereby limiting their use in clinical studies (Table 1).

In a study by Choi et al., genetically labeled RAW 264.7 cells with enhanced firefly luciferase (effluc), referred to as Raw/effluc, were used. The study visualized the intravenously injected Raw/effluc cells in the murine colon (CT26) tumor-bearing mice using bioluminescent imaging in vivo. The findings demonstrated increased prostate cancer targeting of the genetically engineered Raw/effluc cells, resulting in increased tumor size. Consequently, in mice monitored by bioluminescent imaging, macrophage cells migrated to the colon tumor and transformed into tumor-associated macrophages (TAMs), thereby promoting tumor growth [128]. While the study was restricted to a preclinical setting and requires further validation in human colon cancer samples, it does demonstrate the potential of reporter gene-based methods in tracking TAM dynamics in tumors.

Taken together, bioluminescence imaging offers advantages such as low background signal and reduced false-negative results when imaging macrophages or TAMs. Additionally, it allows for the long-term, longitudinal study of macrophage or TAM behavior without the need for repeated injections [88, 120, 126] (Table 1). Thus, the choice between fluorescence or bioluminescence imaging techniques or a combination strategy depends on the research question, the biological system under study, and the desired imaging parameters (Table 1).

### Magnetic resonance imaging (MRI)

Magnetic resonance imaging, or MRI, utilizes strong magnetic fields and radio waves to generate highly detailed images of the body’s internal structures with exceptional clarity and resolution. Unlike X-rays, CT scans, or PET scans, MRI does not involve ionizing radiation. Instead, it capitalizes on the behavior of hydrogen atoms in the body’s tissues when subjected to magnetic fields(^1^H-MRI). In MRI cell tracking, iron oxide nanoparticles are frequently employed as contrast agents. These nanoparticles contain unpaired electrons that align with the applied magnetic field, creating local magnetic field inhomogeneities. These inhomogeneities affect the relaxation times (T1, T2, and T3*) of nearby hydrogen nuclei, enhancing image contrast and enabling precise cell tracking [5, 129–131]. Superparamagnetic iron oxides (SPIOs), ranging from 50 to 100 nm, and ultra-small paramagnetic iron oxides (USPIOs) with diameters less than 50 nm are widely employed in MRI for cell tracking due to their magnetic properties [132]. However, despite high anatomical resolution, MRI suffers from certain disadvantages. This includes lower sensitivity compared to PET or SPECT, and longer scanning times [133]. Apart from the mentioned shortcomings, MRI also suffers from chemical shift artifacts. These artifacts arise due to differences in the resonance frequencies of hydrogen nuclei in various chemical environments, such as fat and water. The variations in local magnetic fields experienced by protons in these environments cause slight frequency differences, which can distort images and lead to spatial misregistration or signal cancellation [92, 93] (Table 1).

Where optical imaging (fluorescence and bioluminescence) has limited tissue penetration [7, 120, 134], MRI provides excellent tissue permeation, making MRI more suitable for visualizing macrophages and TAMs in deep tissues [7, 129]. Accordingly, in a study by Cho et al., peritoneal macrophages from BALB/c nude mice were evaluated for viability, phagocytotic capacity, and migratory activity using the MRI. The T2* of labeled macrophages was assessed using a clinical 1.5 T MR scanner. Specifically, the authors induced metastatic lymph nodes (LNs) in the nude mice and intravenously administered 2 × 10^6^ macrophages labeled with 50 mg Fe/mL ferromagnetic iron-oxide nanocubes (FIONs). After one day, 3D T2*-weighted gradient-recalled-echo MR images were acquired, and the percentage of pixels below the signal intensity threshold was recorded as FION hypointensity. Though the study did not differentiate between the M1 or M2 categories of macrophages in their results, nor did it address the time-dependent changes in the injected macrophages, it did observe that the FION-labeled macrophages targeted the primary tumors and LN metastases (12% FION-macrophage hypointensity compared to 2% of FIONs alone). Thus, the study implied that macrophages could be clinically helpful in delivering therapies to both tumors and LN metastases [135] (Table 2), although the clinical translation of this method remains underexplored.

As described earlier, TAMs encompass diverse subtypes, including M2 macrophages, which are known to fuel tumor growth and metastasis through proangiogenic and growth factor secretion. Consequently, M2 macrophage depletion has been widely explored as a novel anticancer strategy [136], for instance, in a study by Melancon et al. This study crafted a dual magneto-optical probe, PG-Gd-NIR813, to aid in the noninvasive visualization of TAMs after intravenous injection. In rats with C6 tumors, PG-Gd-NIR813 showed maximum tumor uptake at 48 h (Table 2), as confirmed by in vivo/ex vivo optical imaging and T1-weighted MRI. The probe accumulated in necrotic tumor regions and was reduced upon macrophage depletion achieved by clodronate liposomes. Furthermore, immunostaining linked PG-Gd-NIR813 with TAM markers CD68, CD163 and CD169. However, the probe didn’t efficiently differentiate between the tumor-infiltrating monocytes, as indicated by the CD68 staining, which is a macrophage/monocyte marker. Moreover, monocytes also express CD163 and CD169 during inflammation [137, 138], which further underscores the probe’s limitation. Nonetheless, the technique highlights PG-Gd-NIR813’s potential for imaging antitumor responses and as a carrier for immunotherapeutic targeting TAMs [116].

In another study by Agighi et al., 20 patients (10 lymphoma, 10 bone sarcoma) were examined using ferumoxytol-enhanced MRI 24–48 h after injection, followed by tumor biopsy/resection and macrophage staining. The potential of ferumoxytol-enhanced MRI to distinguish tumors with different TAM content was evaluated by comparing the T2* relaxation times of lymphomas and bone sarcomas. Strikingly, the tumor T2* values positively correlated with the CD68^+^ (*r=*-0.68, *P =* 0.031) and CD163^+^ (*r=*-0.76; *P =* 0.010) TAM quantities, as observed by histopathology. Although the authors utilized CD68^+^ and CD163^+^ markers for TAMs, which lack a macrophage-specific expression [137, 138]. Additionally, both bone sarcomas and lymphomas displayed different MRI enhancements and TAM density (*P <* 0.05). Now, whether these differences existed due to differences in the tumor composition or TAM phenotypes (M1 or M2) is not commented upon. Nonetheless, the study provides clinical evidence of ferumoxytol-enhanced MRI as a useful method for categorizing patients with TAM-rich tumors for immune-targeted treatments and tracking therapy responses [139].

In another study, the iron-oxide nanoparticle uptake was compared between F4/80- mammary carcinoma cells and F4/80^+^ TAMs. Remarkably, TAMs phagocytosed iron-oxide nanoparticles more effectively (*d*R2 TAM > *d*R2 cells; *P <* 0.05) than the tumor cells in vitro. The observed MRI enhancements at 1 and 24 h post-injection correlated with TAM presence and were hindered upon TAM depletion by the CSF1 antibody. These results suggest that TAM-mediated uptake of contrast agents acts as a primary source of MRI signal enhancement and could serve as a new biomarker for prognosis, treatment guidance, and immune-targeted therapy assessment for breast cancer [140]. However, the effects of the probe uptake on the TAM phenotype and whether this uptake is specific to a particular subset of TAM remain unexplained. A similar correlation between TAMs and the MRI-iron oxide probe uptake was reported by Leftin et al. In their study, the authors characterized TAMs by analyzing iron distribution on MRI with and without the administration of dextran–ultra-small superparamagnetic iron oxide (USPIO) in orthotopic MMTV-PyMT murine mammary tumors. The specific experimental details are summarized in Table 2. Notably, the + USPIO group showed increased clusters of iron deposits at the tumor’s outer edges compared to the − USPIO group. This correlated positively with a higher frequency of iron^+^CD68^+^ and iron^+^CD206^+^ in the + USPIO group than in the − USPIO group. While these results cannot be generalized to different tumor types due to the heterogeneity of the TME and the TAM composition, the authors suggested that spatial iron deposit distributions rather than the average of the region of interest enhance TAM characterization in breast cancer models [141].

Intriguingly, despite the many advantages of ¹H-MRI, its relatively low sensitivity has driven the development of various contrast agents. The introduction of a “second color” MRI method has addressed several limitations associated with traditional contrast agents, such as the need for pre-scans and the potential for artifacts caused by localization ambiguity. This advancement enhances the ability to distinguish between different tissue types and improves overall imaging accuracy. In this context, ¹⁹F-MRI strategies, alongside ¹H-MRI, have garnered significant attention. The advantages of ¹⁹F atoms include their absence in the human body and the linear relationship between ¹⁹F concentration and MRI signal, which enables quantitative analysis, compared to SPIO cell tracking which is semi-quantitative in nature. However, to achieve an adequate signal-to-noise ratio, high concentrations of ¹⁹F- based contrast agents may be necessary, which can lead to increased cytotoxicity in vivo. This trade-off between signal strength and potential toxicity remains a challenge in the application of ¹⁹F- based contrast MRI for in vivo imaging. Moreover, the stability of labeled nanoemulsions within the body, including their metabolism and clearance rates, needs to be carefully monitored to ensure reliable tracking over time [94–96]. Nonetheless, given the high sensitivity and specificity of ¹⁹F- based contrast MRI, it is often employed in understanding TAMs in multiple tumor models. Makela et al. employed ¹⁹F- based contrast MRI to assess the density and distribution of macrophages within murine breast cancer tumors and associated metastases in vivo. The study involved implanting three murine breast cancer cell lines with varying metastatic potentials (4T1, 168FARN, and 67NR) into the mammary fat pads of mice. In vivo whole-body ¹⁹F- based contrast MRI was performed on tumor-bearing mice 24 h after the intravenous injection of a perfluorocarbon (PFC) agent, which was selectively taken up by macrophages in situ. The results showed that tumor-associated macrophages (TAMs) were predominantly located in the periphery of primary tumors, with higher TAM numbers detected in the more aggressive 4T1 tumors. Interestingly, tumors exhibited significantly greater ¹⁹F signal intensity (spins/mm³) when smaller, suggesting increased TAM infiltration in early-stage tumors. Additionally, ¹⁹F signals were observed in lung metastases of 4T1 tumor-bearing mice, and fluorescence microscopy confirmed the presence of PFC-positive macrophages. These findings indicate that ¹⁹F-MRI can effectively detect and monitor TAMs in individual tumors, enabling the identification of tumors with substantial TAM infiltration. This technique could serve as a potential biomarker for tumor characteristics and might be applicable to other tumor types as well [142]. A recent study by Croci et al. utilized ¹⁹F- nanoparticle MRI to noninvasively track TAMs in glioma models. The authors intravenously injected ¹⁹F-PFC-containing nanoparticles (NPs) into tumor-bearing mice, successfully tracking TAMs over time and in response to radiotherapy. Additionally, they employed multispectral MRI with two different ¹⁹F-PFC-NPs to identify spatially and temporally distinct TAM niches in radiotherapy-recurrent murine gliomas. This approach enabled a deeper understanding of the dynamic behavior and distribution of TAMs in the context of tumor recurrence and treatment response [143].

Thus, MRI-based TAM characterizations hold significant implications in the realm of nanoparticle-enhanced macrophage imaging in cancer research. This is primarily due to the increased spatial resolution and contrast that MRI offers over optical imaging methods, as summarized in Table 1.

#### Magnetic resonance spectroscopy (MRS)

Magnetic Resonance Spectroscopy (MRS) is a non-invasive imaging technique that complements standard MRI by analyzing the biochemical composition of tissues. While MRI provides detailed anatomical information, MRS focuses on measuring the concentration of specific metabolites within cells or regions of interest, offering deeper insights into cellular metabolism, pathology, and disease progression. MRS detects signals emitted by nuclei such as hydrogen (¹H), phosphorus (³¹P), or carbon (¹³C) when exposed to a magnetic field. These signals arise from variations in the chemical environment, known as “chemical shifts,” which result in distinct peaks on the MRS spectrum [97, 98]. Table 1 provides an overview of the key features of MRS, along with its advantages and limitations.

Given its ability to assess physiological status, MRS has been utilized to investigate metabolic differences between M1 and M2 macrophages [99, 100]. However not many studies have employed MRS to investigate the physiology and detect the composition of TAMs in the TME. Nonetheless, a few studies, in combination with MRI, have incorporated MRS into multi-modal imaging strategies for macrophages. Liu et al. developed MRI probes designed to assess nitric oxide (NO) in macrophages, enabling real-time monitoring of macrophage phenotypic changes within tumors. Arginine metabolism differs between macrophage phenotypes, with M1 macrophages producing NO and M2 macrophages generating urea [144]. During tumor treatment, the phenotypic shift from M2 to M1 macrophages involves increased expression of inducible NO synthase (iNOS), resulting in the intracellular production of NO from arginine [145]. To exploit this mechanism, the authors created a NO-responsive nanoprobe based on ultrasmall superparamagnetic iron oxide nanoparticles. This molecular imaging nanoprobe was specifically designed to quantify macrophage repolarization by targeting the redox-active NO as a chemical marker. Equipped with O-phenylenediamine groups, the nanoprobe reacted with intracellular NO during the M2-to-M1 transition, triggering electrical attraction and colloidal aggregation of the nanoparticles. These structural changes lead to significant alterations in T1 and T2 relaxation times in MRI, allowing precise quantification of macrophage polarization. In a 4T1 breast cancer model, this MRI nanoprobe effectively visualized macrophage polarization and predicted treatment outcomes in immunotherapy and radiotherapy settings [146]. Future research could explore the applicability of this strategy across other tumor models, broadening its clinical potential. Moreover, identifying key metabolic differences between TAM subtypes could further enhance the design of MRI-based probes, enabling more precise targeting and monitoring of macrophage polarization within the tumor microenvironment.

#### Magnetic particle imaging (MPI)

Magnetic Particle Imaging (MPI) is an advanced imaging technology that directly detects magnetic nanoparticles (MNPs), such as iron oxide, with high sensitivity and non-invasiveness. This modality uses superparamagnetic iron oxide nanoparticles (SPIONs) as tracers, which exhibit no remanent magnetization after the removal of the magnetic field, making them particularly suitable for imaging applications. The technique applies a spatially varying magnetic field to the imaging region, creating a field-free region (FFR) or field-free point (FFP). When MNPs are exposed to oscillating magnetic fields, they produce harmonics in their response signal. The scanner detects only these nonlinear signals from the MNPs, effectively ignoring background signals from surrounding tissues or structures, resulting in high specificity [101–103].

While MRI remains the gold standard for high-resolution, whole-body imaging and superior soft-tissue contrast, the high sensitivity of MPI in detecting even minute concentrations of MNPs, coupled with its ability to avoid background noise, has made it an increasingly popular tool for analysing TAMs in the TME in preclinical studies (Table 1). The technique was first employed by Yu et al. for in vivo cancer imaging using systemically administered tracers. They developed long-circulating, MPI-tailored SPIOs, which were intravenously injected into tumor-bearing rats. The tumors were distinctly highlighted, achieving a tumor-to-background ratio of up to 50. Additionally, the nanoparticle dynamics within the tumor were well characterized, showing an initial wash-in at the tumor rim, peak uptake at 6 h, and eventual clearance beyond 48 h. Although the authors did not investigate whether tumor-associated cells, such as TAMs, contributed to the observed signal, the study effectively demonstrated the quantitative capabilities of MPI using compartmental fitting in vivo [104]. In another study, Makela et al. compared the detection capabilities of MRI and MPI for iron-labelled macrophages associated with cancer. In their study, imaging was conducted on 4T1 tumor-bearing mice 16–21 days post-cancer cell implantation and 24 h after intravenous administration of either Ferucarbotran, a superparamagnetic iron oxide (SPIO), or Ferumoxytol, an ultra-small SPIO. Living mice were imaged using a 3T clinical MRI system (General Electric, *n* = 6) and an MPI system (Magnetic Insight, *n* = 10). Following imaging, tumors and lungs were excised, further imaged using MPI, and analysed through histology. The study concluded that MPI provides quantitative in vivo data on iron labelling of macrophages, a level of information unattainable with MRI. Additionally, Ferumoxytol nanoparticles outperformed Ferucarbotran in enabling the MPI-based detection of macrophages labelled in vivo [105]. Interestingly, in recent years there has also been advancements in generating more optimized SPIO and USPIO for MRI/MPI, which perform better than ferumoxytol, like ferucarbotran as depicted by the study mentioned above [147]. These include gadolinium, iron and manganese-based agents (Table 1) [148], which overcome the shortcomings of ferumoxytol.

Although limited studies have specifically investigated TAM phenotypes and behaviour within tumors using MPI, the technique holds significant future potential. For example, due to its quantitative capabilities, MPI could be utilized for macrophage-mediated tumor therapy and detection. In a notable study, Zu et al. designed a superparamagnetic Fe_3_O_4_ nanocluster@poly(lactide-co-glycolide acid) core–shell nanocomposite loaded with the chemotherapy drug doxorubicin. This innovative system served as both a drug delivery platform and an MPI quantification tracer. The nanocomposite’s degradable nature in a mildly acidic microenvironment (pH = 6.5) facilitated sustained doxorubicin release and gradual decomposition of the Fe_3_O_4_ nanocluster, leading to measurable changes in the MPI signal. The study demonstrated a strong linear correlation (R² = 0.99) between MPI signal changes and the doxorubicin release rate over time. Furthermore, in a murine breast cancer model, they monitored drug release and its therapeutic effect on tumor cells through MPI, highlighting the feasibility of in vivo drug release tracking in a cancer therapy context [149]. Future research could focus on leveraging such nanocomposite systems to deliver MPI tracers alongside therapeutic agents within macrophages. This approach could not only enhance tumor cell killing through combined drug action and macrophage-intrinsic tumor-clearing mechanisms but also enable real-time tracking of macrophage dynamics and efficacy in tumor clearance. Such advancements could pave the way for improved preclinical and clinical applications of MPI in cancer therapy.

### Nuclear imaging: positron emission tomography (PET) and single-photon emission computed tomography (SPECT)

PET scanning involves measuring the concentration of positron-labeled molecular probes, like ligands or substrates, attached to specific target proteins or confined within cells of interest. In contrast, SPECT imaging agents are labeled with γ-emitting radionuclides (e.g., technetium-99m (^99m^Tc), iodine-123 (^123^I), and iodine-131 (^131^I)), whereas PET tracers utilize positron-emitting radionuclides (e.g., fluorine-18 (^18^F), iodine-124 (^124^I), and copper-64 (^64^Cu)) [5]. Unlike MRI and optical imaging methods described above, PET and SPECT require only small quantities of imaging agents (ranging from nanograms to milligrams) [133]. Consequently, radionuclide-based imaging agents employed in PET or SPECT studies are generally safe, and involve a small amount of radiation, which unlikely to induce pharmacological effects; The radiation dose during scanning must be carefully managed to avoid disrupting the biological system and causing toxicity, PET and SPECT studies use very small amounts of tracers, which generally not produce pharmacological effects [133, 150, 151].

Like MRI, nuclear imaging methods offer the advantage of deep tissue penetration, making them suitable for imaging macrophages or TAMs in deep-seated tumors [152]. Since PET/SPECT primarily provides 3D functional information about the tissue of interest with relatively poor resolution, therefore, combining it with MRI or CT, which offers high-resolution structural details, creates a powerful hybrid imaging modality. This integration enables simultaneous acquisition of functional/metabolic and anatomical/structural information, enhancing diagnostic accuracy and utility [111] (Table 1). Nonetheless both, PET and SPECT alone or in combination with MRI is widely used to track TAMs in therapies. For instance, Lee et al. investigated the delivery of photothermal therapy (PTT) using radioiodine-labeled gold nanoparticles (^124^I-Au@AuCBs) loaded onto macrophages (Raw 264.7). These ^124^I-Au@AuCB-labeled macrophages were then intratumorally injected into murine colorectal carcinoma (CT26/FM) tumors, followed by PET/CT scans (Table 2), which revealed an even distribution of the probe within the tumor lesions. Upon NIR laser irradiation (6.0 W cm^− 2^, 808 nm) to the tumor site, the authors reported potent antitumor effects. While the impact of transferred macrophages on the host immune system and long-term effects remain unaddressed, these findings underscored the potential of ^124^I-Au@AuCBs theranostic materials, highlighting the benefits of macrophage-mediated drug deliveries coupled with PET/CT for various conditions, including cancer [153].

Apart from tracking therapeutic macrophages, nuclear imaging techniques are often used to track the behavior of TAMs within the TME. Pérez-Medina et al., two different radiolabeled reconstituted high-density lipoprotein (rHDL) nanoparticles containing ^89^Zr (called ^89^Zr-PL-HDL and ^89^Zr-AI-HDL), were intravenously injected into mice bearing the orthotropic breast (4T1) tumors. Quantitative PET imaging and histological analysis, which included ionized calcium-binding adaptor molecule 1 (Iba-1) as a macrophage marker [154], demonstrated that ^89^Zr-PL-HDL displayed significant colocalization with macrophage-rich regions. However, the study also noted an uptake by a cell subtype referred to as monocyte-derived cells. Consequently, while the technique did not sufficiently separate different immune cell types that take up the probe in tumors nor differentiate between M1 or macrophage M2 subtypes, the use of the ^89^Zr-rHDL imaging agents for quantitative macrophage PET imaging exhibited substantial potential in noninvasively monitoring the complex behaviors, functional states, and interactions of macrophages within the TME [155]. Coupling macrophage-specific antibodies to PET/CT probes has gained importance due to the specificity it offers. In a previous study, single-domain antibody fragments of the cross-reactive antimacrophage mannose receptor (MMR, CD206), a C-type lectin receptor on M2 macrophages that plays a critical role in pathogen recognition, antigen presentation, and immune regulation [156, 157], were linked to the PET tracer ^18^F-fluorobenzoate (^18^F-FB). The ^18^F-FB-antiMMR was injected into mouse lung carcinoma (3LL-R) tumor-bearing mice (both wild type and MMR-deficient), followed by PET imaging at 3 h. (Table 2). Notably, there was significant tumor-specific retention in the wild-type mice (3 times higher mean uptake) compared to the MMR-deficient mice. This confirmed the specificity of the ^18^F tracer specificity for MMR and TAMs, respectively, highlighting its potential for precise macrophage imaging in patients [158]. However, the use of short half-life radionuclides (e.g. ^18^F) in PET/CT makes such approaches unsuitable for longitudinal studies (Table 1).

Another study by Kim et al. developed a poly-glucose nanoparticle labeled with ^64^Cu (referred to as Macrin) for tracking TAMs using a quantitative PET imaging approach. Macrin and its analogs were injected intravenously into mice bearing murine colon adenocarcinoma (MC38) tumor, a TAM-rich model [12]. PET/CT imaging was acquired 24 h after injection (Table 2), revealing high Macrin accumulation in the cancer. Furthermore, Macrin imaging was utilized to monitor the response of macrophages to chemotherapy and γ-irradiation treatments. While the clinical relevance of Macrin requires validation and potential side effects need assessment in patients, it presents a promising selective and translational method for quantifying TAMs and guiding therapeutic decisions [159].

SPECT imaging is also utilized to track TAMs within the TME in combination with macrophage-specific antibodies. For instance, Zhang et al. developed M2-targeted probes for SPECT imaging using an anti-CD206 monoclonal antibody to track TAMs within the TME. They evaluated the specificity and potential applications of these probes in murine breast (4T1) tumor models, including subcutaneous tumors and LN metastasis. Following cyclophosphamide treatment, the authors observed a significant increase in M2 macrophage infiltration in relapsing 4T1 tumors but not in non-relapsing ones. Using the synthesized SPECT probes, they sensitively detected M2 macrophage infiltration in relapsing tumors and LN metastasis. Importantly, early tumor relapse prediction through the molecular imaging of M2 macrophages enabled effective tumor eradication when combined with radiotherapy. Therefore, M2 macrophage–targeted imaging facilitates noninvasive prediction of post-chemotherapy tumor relapse and sensitive detection of metastatic LNs in vivo, offering insights into cancer progression, early resistance prediction, and implications for cancer therapeutics [112].

In another study, mannosylated liposomes (MAN-LIPs) were utilized, which specifically accumulated in TAMs in a mouse model of pulmonary adenocarcinoma. These liposomes contained ^64^Cu for PET imaging, followed by microscopy analysis. Interestingly, MAN-LIPs predominantly gathered in TAMs with minimal accumulation in distant lung areas at 6 h. after injection (Table 2). This study demonstrates that MAN-LIPs offer a promising approach for delivering imaging agents to lung TAMs and potentially for therapeutic agent delivery to the TME [160]. However, the clinical implications of this approach and the long-term effects of the probe on TAM phenotype and function require further investigation.

Tumor hypoxia and aerobic glycolysis are known resistance factors in cancer treatment. Apart from explicitly targeting TAMs using MMR, this marker can also be utilized to differentiate the targeting of TAM subsets within the TME. Interestingly, TAMs located in hypoxic regions and exhibiting elevated MMR expression play a substantial role in shaping the TME due to their strong proangiogenic characteristics. A study by Movahedi et al. utilized MMR-specific nanobodies (Nbs) labeled with ^99m^Tc for in vivo TAM imaging. The ^99m^Tc-labeled a-MMR Nb cl1 was injected intravenously in lung carcinoma (TS/A and 3LL-R) tumor-bearing mice, and SPECT imaging was performed 1 h after injection (Table 2). Both TS/A and 3LL-R tumors exhibited substantial uptake of the ^99m^Tc-labeled a-MMR Nb cl1 in WT animals (2.02 ± 0.11), compared to the MMR-deficient mice (0.06 ± 0.01). Histology revealed that within tumors, the labeled Nbs were specifically labeled as MMR-positive TAMs (Ly6C^int^ MHCII^lo^). Notably, the anti-MMR Nbs accumulated in hypoxic regions, precisely targeting proangiogenic MMR-positive TAMs. Taken together, anti-MMR nanobodies can be utilized to target selectively and image TAM subpopulations in vivo and study metabolic reprogramming in tumors [161] (Table 1).

As stated above, TAMs preferentially localize in hypoxic and metabolically distinct regions of the TME, further exacerbating these conditions. For instance, Jeong et al. reported a strong correlation between CD68^+^ TAM immunostaining and ^18^F-fluoro-deoxyglucose (FDG) uptake on PET imaging (FDG SUVmax; rho = 0.369; *P <* 0.001 and 40% TLG uptake; rho = 0.355; *P <* 0.001) in non-small cell lung cancer (NSCLC). The authors suggested that TAMs increase tumor cell glycolysis by secreting TNFα and elevate tumor hypoxia by enhancing AMP-activated protein kinase and PPARγ coactivator 1-α. Furthermore, depletion of TAMs not only eliminated tumor-associated hypoxia but also increased PD-L1 expression on cancer cells and T-cell infiltration, making PD-L1 antibodies more effective. Therefore, TAMs can significantly impact tumor metabolism and complicate responses to anticancer therapies, including immunotherapy [162]. Although the depletion of TAMs in patients is challenging, the study indicates the potential of developing combination therapies for cancer patients.

**Fig. 3:**
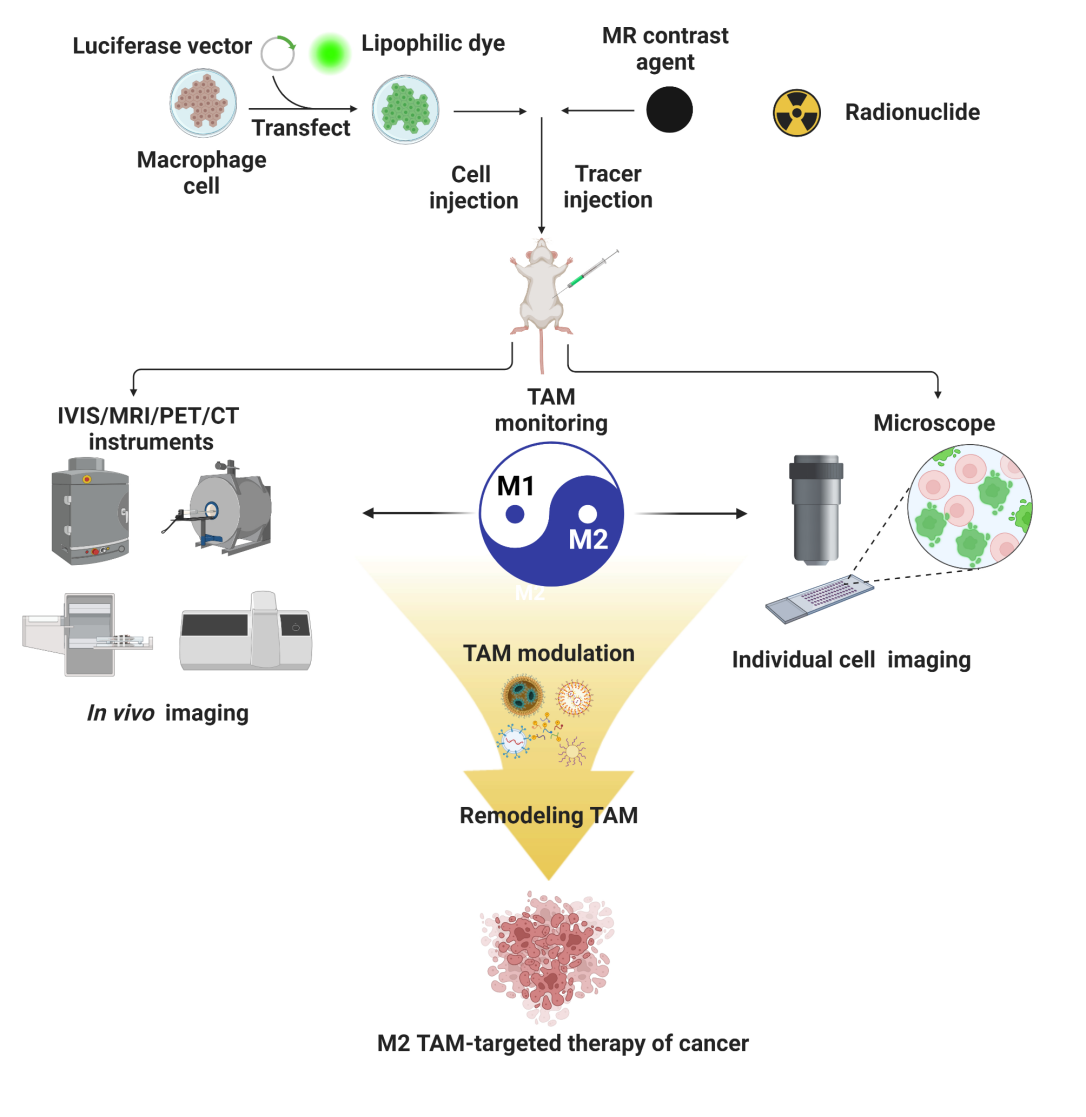
Noninvasive in vivo imaging of macrophages and TAMs. TAM-directed imaging and therapy are interdependent and can improve clinical outcomes. In vivo imaging such as optical in vivo imaging system (IVIS), PET/CT, and microscopic analysis can be helpful to monitor and track down the TAM population in tumors noninvasively. In addition, TAM-directed imaging can be designed to target macrophages with drugs, leading to the modulation of TAM, thereby resulting in combined diagnostic and therapeutic effects for better outcomes. Created with BioRender.com

Figure 3; Table 2 offer an overview of research efforts dedicated to visualizing and monitoring macrophage migration and infiltration in the context of tumor biology, showcasing the diverse imaging techniques and their applications in this field.

## In vivo molecular imaging of macrophage-derived extracellular vesicles in targeting tumors: insights from fluorescence imaging modality

Understanding the fate of macrophage-derived EVs within living organisms, particularly their propensity to accumulate at tumor sites, is crucial for optimizing EV derivation and administration protocols to improve their therapeutic efficacy. EVs, in general, face challenges such as rapid clearance by the body, which limits their ability to target tumors effectively [163–166]. Moreover, given these limitations, the clinical translation of macrophage-EV-based therapies remains limited, with most studies still at the preclinical stage. Intriguingly, fluorescence imaging is commonly used to track macrophage-EVs in vitro and in vivo, while other modalities remain underexplored. This reliance on fluorescence imaging underscores the need for further research into alternative imaging techniques that could enhance the tracking and efficacy of macrophage-EV-based therapies.

To address the issue of EV-tumor targeting, various strategies have been developed to monitor and enhance macrophage-EV targeting for therapeutic purposes. A study by Baek et al. demonstrated that proinflammatory macrophage (M1)-derived nanovesicles (M1-NVs) can boost antitumor effects through a surface modification technique using polyethylene glycol (PEG). The authors labeled both the bare M1-NVs and PEG- M1-NVs with DiR dye and injected them intravenously into colon cancer-bearing mice. They generated M1 macrophages through LPS/IFNγ treatment of RAW 264.7 cells, a method that could modify macrophage behavior but was not addressed in the study. Nonetheless, in vivo fluorescence imaging showed that PEG- M1-NVs targeted the tumor within 3 h., while no signal was detected in the tumors of mice injected with bare M1-NVs, even after 24 h. (Table 3). These results indicate that PEG significantly enhances M1-NVs’ ability to target tumors in vivo, potentially advancing EV-based therapeutics for various diseases, including cancer [167]. However, the generation of M1 cells in vitro and the costly nature of exosome isolation limit the method’s translational potential.

**Table 3:** In vivo molecular imaging of macrophage-derived extracellular vesicles in targeting tumors

Imaging	Imaging modality	Labeling Agent	Cell Source	EVs type	Subject	Injection route	Duration	Tumor	Ref.
FLI	FLI	DiR	RAW 264.7 cells	M1-NVs	BALB/c mice	IV	0, 3, 12, & 24 h	Mouse colon cancer	[167]
	FLI	DiD	Primary macrophages	M1-Exosome	BALB/c mice	IV	2 h	Mouse breast cancer	[67]
	FLI	Cy5.5	RAW 264.7 cells	Exosome	BALB/c nude mice	IV	4, 8, 12, & 24 h	Human breast cancer	[168]
	FLI	Cy5	THP1 cells	Exosome	BALB/c nude mice	IV	4, 8, 12, & 24 h	Human breast cancer	[169]
	FLI	PpIX	RAW 264.7 cells	EVs	BALB/c mice	IV	4, 8, 12, 24 & 48 h	Mouse breast cancer	[170]

FLI - Fluorescence Imaging; DiR − 1,1’-Dioctadecyl-3,3,3’,3’-Tetramethylindotricarbocyanine Iodide; DiD − 1,1’-Dioctadecyl-3,3,3’,3’-Tetramethylindodicarbocyanine; PpIX: Protoporphyrin IX; M1-NVs - M1 Macrophage-derived Nanovesicles; I.V – Intravenous

Another study that utilized fluorescence imaging to track macrophage-EV-based therapy comes from Li et al., who developed a novel approach using macrophage-derived exosomes coated with poly (lactic-co-glycolic acid) for targeted chemotherapy of triple-negative breast cancer (TNBC). TNBC, the most aggressive breast cancer subtype, lacks effective therapeutic targets, leaving chemotherapy and surgery as the primary options. Yet, chemotherapy faces limitations like poor targeting and high toxicity. In vivo, Cy5.5 labeled PL-D (DOX loaded PLGA nanoparticles), EP-D (macrophage exosome coated nanoparticle), and MEP-D (c-Met binding peptides were decorated on the macrophage exosome) were administered via the tail vein to nude mice with MDA-MB-231 tumors. After 4 h., MEP-D exhibited robust fluorescence at the tumor site, indicating its notable targeting efficacy. Notably, MEP-D showed consistently higher fluorescence intensity at the tumor site compared to PL-D or EP-D at various time points up to 24 h. These findings suggest that MEP-D demonstrates a strong ability to target tumors [168]. However, similar to the study by Baek et al., this approach requires further validation in pre-clinical settings to establish its translational potential. Apart from targeting tumors, macrophage-derived EVs have also been utilized to deliver drugs to tumor sites for TNBC. For instance, Gong et al. developed a strategy to enhance exosome binding to integrin αvβ3 by creating A15-modified Exo (A15-Exo) for precise co-delivery of doxorubicin (Dox) and cholesterol-modified miRNA 159 (Cho-miR159) to TNBC cells. Fluorescence imaging showed that 2 h. after IV administration (Table 3) of free Cy5-Cho-miRNA, Exo-Cy5-Cho-miRNA, or A15-Exo-Cy5-Cho-miRNA in nude mice with MDA-MB-231 tumors, there was a substantial accumulation of Cy5 fluorescence in the tumor areas in the A15-Exo-Cy5-Cho-miRNA group, contrasting with liver or kidney accumulation in other groups. Further results demonstrated that A15-Exo-Cy5-Cho-miRNA efficiently suppressed tumor growth and enhanced the survival rate of mice with tumors [169].

A study by Li et al. coupled tumor targeting with therapy by employing a straightforward method to encapsulate folate (FA)-modified EVs for targeting tumors. These EVs were loaded with the photosensitizer protoporphyrin IX (PpIX) and doxorubicin (Dox) for therapeutic outcomes. Macrophages effectively convert 5-aminolevulinic acid (5-ALA) into protoporphyrin IX (PpIX) through the mitochondrial heme synthesis pathway. Therefore, macrophages were incubated with Dox, 5-ALA, and DSPE-PEG-FA in the culture medium. The macrophages then secreted extracellular vesicles (EVs) with surface modifications targeting tumors through folate (FA) while also encapsulating biosynthesized PpIX and Dox, referred to as PpIX-DOX@FA-EVs. Free PpIX, PpIX-DOX@EVs, and PpIX-DOX@FA-EVs were intravenously injected into mice bearing 4T1 tumors (Table 3). Fluorescence imaging revealed that PpIX-DOX@FA-EVs swiftly gathered at the tumor location within 4 h. post-injection, with notably lower concentrations observed in healthy organs such as the heart, liver, lungs, and kidneys. This demonstrates the efficient delivery of therapeutic molecules to tumor sites, achieving effective anti-cancer treatment [170].

Table 3 offers an overview of research efforts dedicated to visualizing and monitoring macrophage-derived EVs migration and infiltration in the context of tumors. Notable EV studies in clinical trials highlight the diverse therapeutic potential of EVs across various applications, including cancer therapy, neuroprotection, ischemic recovery, and regenerative (Table 4). These studies demonstrate the translational capabilities of EVs, showcasing their roles in targeted immunotherapy, drug delivery and tissue repair. By leveraging these insights, the translational pathway for macrophage-derived EVs can be further advanced, paving the way for their development into clinical applications. Further research into macrophage-derived EV visualization is imperative to unlock their full potential in both basic science and clinical applications.

**Table 4:** Extracellular vesicle (EV) studies in clinical trials and their translational potential

NCT	Type of EVs	Disease	Expected Outcomes or Outcomes	Ref.
NCT01779583	Dendritic Cell-derived Exosomes (Dex)	NSCLC	• Phase I trials showed the safety and feasibility of Dex vaccines. • Phase II trial confirmed the capacity of Dex to boost the NK cell arm of antitumor immunity.	[172, 173]
NCT05375604	exoASO-STAT6 (CDK-004)	Advanced HCC and Liver Metastases (Primary Gastric Cancer or Colorectal Cancer)	• Dose escalation, safety, pharmacodynamic, and PK study.	[174]
NCT04388982	Allogenic Adipose MSC-Exos	Alzheimer’s disease	• MSCs-Exos was safe and well tolerated. • Recommended dose of at least 4 × 10^8^ particles for further clinical trials.	[175, 176]
NCT03384433	Allogenic Placenta MSC-Exos	Acute Ischemic Stroke	• No post-interventional adverse effects.	[177, 178]
NCT05060107	MSC-Exos	Knee Osteoarthritis	• Study the safety and any adverse Event.	[179]

Exos – Exosomes; Dex - Dendritic Cell-derived Exosomes; MSC - Mesenchymal Stem Cells; NSCLC - Non-small-cell lung cancer lung carcinoma; HCC - Hepatocellular Carcinoma

## Clinical implication

In applying and evaluating macrophage efficacy in humans, tracing and localizing the macrophages are essential. Several studies have assessed the macrophages in humans [139, 162], highlighting their significance and potential therapeutic interventions. These studies have utilized advanced imaging techniques for diagnosis and monitoring of macrophage distribution, behavior, and response to treatments, providing valuable insights into their roles and potential as therapeutic targets in clinical settings (Table 5). For instance, ferumoxytol, developed initially as an iron supplement, can also be used as a T2 MRI contrast agent. Given macrophages can engulf that ferumoxytol, it effectively demonstrates the quantity of TAMs [140, 180]. This dual functionality makes ferumoxytol a valuable tool for both therapeutic and diagnostic purposes, providing insights into macrophage presence and activity in TME. Indeed, Aghighi et al. conducted a ferumoxytol-enhanced MRI post-contrast versus pre-contrast (*P =* 0.036) in 25 patients, including 12 with lymphoma and 13 with bone sarcoma. They demonstrated T2* signal enhancement on MR images correlated significantly with the density of CD68^+^ and CD163^+^ TAMs (*P <* 0.05) [139]. However, whether this approach can be extended to studying other tumor types remains controversial. Intriguingly, ferumoxytol has been associated with MRI artifacts in the brain [181, 182], which limits its utility in understanding TAMs in brain tumors. Additionally, ^18^F-FDG PET/CT is the most popular radiotracer for representing cellular glycolysis. Jeong et al. demonstrated a strong correlation between CD68^+^ TAM immunostaining and ^18^F-FDG PET/CT uptake in 98 matched tumors of patients with NSCLC [162]. Indeed, while both ^18^F-FDG PET/CT tracers correlated well with TAM density, they are not specific for TAMs and have not been tested clinically. As of now, FDA-approved specific agents for in vivo macrophage imaging in humans are still limited. Hence, there is a pressing need for the development of more specific agents tailored to target TAMs selectively.

**Table 5:** Macrophage-based tumor targeting strategies and in vivo imaging modalities in recent clinical trials

Trial ID	Macrophage/tumor target	Cancer type	Imaging modality used	Drug used	Reference
NCT03242993	Folate receptor on TAM	Metastatic lung and ovarian cancer	PET [^18^F]AzaFol	Folarell	[185]
NCT03608618	Tumor cell mesothelin; CAR-M	Ovarian cancer and peritoneal mesothelioma	Fluorescence imaging (in preclinical model)	Cyclophosphamide	[186]
NCT06224738	Tumor cell-associated Her-2; CAR-M	Gastric cancer	Fluorescence imaging (in preclinical model)	None	[187, 188]
NCT05933239	Mannose-receptor expressing TAM	Non-small cell lung cancer	PET/CT	^68^GaNOTA-Anti-MMR-VHH2	[189]
NCT01336803	CD68^+^ CD163^+^ TAMs (diagnostic)	Bone sarcoma Osteomyelitis	MRI	Feraheme (Ferumoxytol)	[139, 190]
NCT01542879	CD68^+^CD163^+^ TAMs (diagnostic)	Pediatric solid tumor	WB-DW-MR/ ^18^FFDG PET	Feraheme (Ferumoxytol)	[139, 191]

CAR- Chimeric antigen receptor-Macrophage; TAM- Tumor-associated macrophage; CAR-M- macrophages with CAR; CD- Cluster of differentiation; MRI- Magnetic resonance imaging; PET- Positron emission tomography; CT- Computed tomography; ^18^F-FDG- ^18^F-Fludeoxyglucose; WB-DW-MR- Whole body diffusion weighted magnetic resonance imaging; MRI- Magnetic resonance imaging; [18F]AzaFol-3′-aza-2′-[18F]fluorofolic acid; MMR- macrophage mannose receptor

Moreover, the limited tissue penetration of optical imaging tracers may pose challenges for clinical translation. However, recent advances have shown the utility of fluorescent imaging for evaluating surgical margins in the surgical field [183, 184]. Given that TAMs are one of the constituents of cancer lesions, achieving precise resection of TAMs could be a method for complete tumor resection [34]. Fluorescent imaging systems thus offer several advantages in the surgical setting and improve surgical outcomes. Table 5 compiles select clinical trials actively employing fluorescent imaging strategy for tracking TAMs and aiding in cancer diagnosis.

## Future perspectives

The future of advances in noninvasive, in vivo macrophage imaging holds tremendous potential in further illuminating TME and advancing cellular-based drug-delivery systems. As technology continues to evolve, several exciting perspectives emerge that could transform cancer research and therapy. Combining different imaging modalities, such as MRI, PET, CT, and optical imaging [112, 160–162], could provide a more comprehensive and nuanced view of macrophage behavior and its interactions within tumors. This multimodal approach would leverage the strengths of each technique, enhancing spatial and temporal resolutions while mitigating their limitations [192, 193]. The development of more sophisticated nanoparticle-based contrast agents and molecular probes has the potential to enable ultra-high-resolution imaging at the molecular level. These agents could be engineered to target specific macrophage subsets or markers, allowing for deeper insights into their roles in various disease states. Such advancements hold promise for unraveling the complexities of the TME and identifying targets for cancer treatment.

The integration of machine learning and artificial intelligence (AI) algorithms could revolutionize macrophage image analysis. These tools could facilitate the identification of subtle changes in macrophage behavior, aiding in the early detection of disease progression and providing insights into treatment responses [194, 195]. Advancements in real-time imaging technologies could offer a dynamic, moment-to-moment view of macrophage behavior within tumors. These developments could lead to a more precise understanding of their responses to therapies, allowing for rapid adjustments in treatment strategies.

As immunotherapies continue to transform cancer treatment, integrating macrophage imaging with immunotherapeutic strategies could unlock new avenues for synergistic therapies. Monitoring macrophage responses could guide the timing and combination of treatments, maximizing their impact on tumor clearance [12, 112, 140, 155, 158, 160]. The ability to track macrophage behavior in real-time could promote personalized treatment regimens. Multimodal imaging enables a customized approach to assessing TAMs. By tailoring the combination of imaging techniques to a patient’s specific tumor characteristics, clinicians can decide and implement optimal treatment strategies, improving patient outcomes while minimizing adverse effects. This integrated approach has the potential to significantly enhance the efficacy of cancer immunotherapies and improve the precision of treatment protocols.

The prospects for advances in noninvasive, in vivo macrophage imaging are up and coming. By harnessing the power of evolving imaging technologies, artificial intelligence, and innovative drug-delivery strategies, our understanding of TME may be deepened, revolutionizing cancer treatment. As these visions become realities, they hold the potential to improve precision medicine significantly, enabling therapies to be tailored to the unique characteristics of each patient’s disease.

## Conclusion

The remarkable advancements in noninvasive, in vivo macrophage imaging have led to a profound understanding of the TME and catalyzed significant developments in cellular-based drug-delivery systems. By visualizing and monitoring macrophage dynamics within these complex ecosystems, crucial insights into the intricate interplay between immune cells and cancer cells have been uncovered. This newfound knowledge has not only deepened our comprehension of tumor progression, immune response, and therapy resistance but has also opened avenues for innovative therapeutic strategies. Moreover, through the development of targeted contrast agents and molecular probes, the specificity and sensitivity of macrophage imaging have enhanced, improving our ability to decipher their multifaceted roles within the TME. Furthermore, the synergy between noninvasive imaging and cellular-based drug-delivery systems have advanced personalized medicine. By harnessing the unique properties of macrophages as carriers, therapeutic payloads can be precisely delivered to tumor sites, minimizing off-target effects while maximizing therapeutic outcomes. The versatility of macrophages in modulating their cargo and response to various stimuli has facilitated the design of multifunctional drug-delivery platforms that can adapt to the dynamic TME. Essentially, the convergence of noninvasive in vivo macrophage imaging and cellular-based drug delivery systems holds great promise in enhancing cancer diagnosis, monitoring, and treatment with high precision and efficacy. By elucidating the intricate interactions within the TME and leveraging the potential of macrophages as therapeutic carriers, we stand at the threshold of a transformative era in cancer research and treatment.

## Data Availability

No datasets were generated or analysed during the current study.
